# ISG12a and its interaction partner NR4A1 are involved in TRAIL‐induced apoptosis in hepatoma cells

**DOI:** 10.1111/jcmm.14251

**Published:** 2019-03-01

**Authors:** Nianli Liu, Zhiyuan Wu, Aoxing Chen, Dafei Chai, Liantao Li, Longzhen Zhang, Junnian Zheng

**Affiliations:** ^1^ Cancer Institute, Xuzhou Medical University Xuzhou Jiangsu China; ^2^ Department of Radiation Oncology Affiliated Hospital of Xuzhou Medical University Xuzhou Jiangsu China; ^3^ Center of Clinical Oncology Affiliated Hospital of Xuzhou Medical University Xuzhou Jiangsu China; ^4^ Jiangsu Center for the Collaboration and Innovation of Cancer Biotherapy Cancer Institute, Xuzhou Medical University Xuzhou Jiangsu China

**Keywords:** apoptosis, ISG12a, liver cancer, NR4A1, TRAIL resistance

## Abstract

Tumor necrosis factor‐related apoptosis‐inducing ligand (TRAIL) can induce apoptosis in cancer cells while sparing normal cells, thereby leading to the development of TRAIL receptor agonists for cancer treatment. However, these agonist‐based therapeutics exhibit little clinical benefits due to the lack of biomarkers to predict whether patients are responsive to the treatment, as well as determine the resistance of cancer cells to TRAIL‐based agonists. Our previous study has demonstrated that ISG12a enhances TRAIL‐induced apoptosis and might serve as a biomarker to predict the TRAIL response. The downstream mechanism by which ISG12a augments TRAIL‐induced apoptosis remains to be elucidated. In this study, we found that ISG12a was localized in the mitochondria and nucleus and augmented TRAIL‐induced apoptosis through intrinsic apoptotic pathway. In addition, ISG12a interacted with NR4A1 and promoted its nuclear‐to‐cytoplasm translocation. Upon translocate to cytoplasm, NR4A1 targeted mitochondria and induced Bcl2 conformational change, thereby exposing its BH3 domain. Moreover, TRAIL treatment can induce NR4A1 expression through the activation of NF‐κB in TRAIL‐resistant Huh7 hepatoma cells. Knockdown of NR4A1 could overcome TRAIL resistance. However, in TRAIL‐sensitive LH86 liver cancer cells, TRAIL activated the Jun N‐terminal kinases signalling pathway. Overall, these results showed that both ISG12a and its interaction partner NR4A1 are involved in TRAIL‐mediated apoptosis in hepatoma cells.

## 1 INTRODUCTION

The tumour necrosis factor (TNF)‐related apoptosis‐inducing ligand (TRAIL) is a member of the TNF superfamily and shares sequence homology with Fas ligand and TNF.^1^ Binding of TRAIL to its functional receptors DR4 and DR5 induces the formation of death‐inducing signalling complex (DISC), thereby resulting in activation of initiator caspase‐8 and caspase‐10.^2^ After activation, caspases‐8 and ‐10 then cleave and activate the executioner caspases‐3 and ‐7, thereby triggering the execution of apoptosis.^3^ Tumour necrosis factor‐related apoptosis‐inducing ligand selectively kills tumour cells but spares normal cells. Thus, it is a potential drug for cancer treatment. However, TRAIL also activates a non‐apoptotic pathway, such as NF‐κB signalling pathway, thereby resulting in resistance to TRAIL‐induced apoptosis.^4^

Interferons stimulated genes (ISGs) are executioners of interferons to manifest their cellular biological activities such as antiviral, anti‐tumour and immunomodulatory functions. Unlike other TNF superfamily members, TRAIL was also recognized as an apoptosis‐related ISG.^5^ IFN‐α induces apoptosis in myeloma cells and in solid tumours through induction of TRAIL.^6^, ^7^ Some studies have reported that ISGs can regulate TRAIL‐induced apoptosis. Knock‐down of Xaf1 in glioblastoma cell lines led to the complete loss of IFN‐β‐mediated TRAIL sensitivity, thereby indicating that Xaf1 was a crucial ISG and played key roles in TRAIL‐induced cell death.^9^ The FAM14 family member G1P3 is another ISG that antagonized TRAIL‐induced apoptosis through the inhibition of intrinsic apoptotic pathway.^10^

ISG12a can be robustly induced by IFNs and belongs to the FAM14 family. Unlike the anti‐apoptotic function of G1P3, ISG12a exerts pro‐apoptotic effects on cancer cells.^11^, ^12^ ISG12a localizes to the mitochondria, nuclear membrane and endoplasmic reticulum in different cell lines.^11^, ^12^, ^14^, ^15^ The different subcellular distributions of ISG12a suggest that it may have diverse biological functions. Upon localization to the mitochondria, ISG12a interacts with Bcl2 and may neutralize its anti‐apoptotic effects by displacing it from bcl‐2‐associated X protein (BAX).^11^ When localized to the inner nuclear membrane, ISG12a interacts with NR4A1 and inhibits its transcription activity.^15^ When NR4A1 translocates from the nucleus to the cytoplasm, it can target the mitochondria, interacts with Bcl2 and converts Bcl2 from an anti‐apoptotic molecule to a pro‐apoptotic molecule.^16^ However, whether the interaction between ISG12a and NR4A1 is involved in TRAIL‐induced apoptosis in hepatocellular carcinoma (HCC) is still unknown.

We previously reported that high expression level of ISG12a was positively associated with TRAIL‐induced apoptosis in HCC cells.^17^ However, the underlying mechanism by which ISG12a augments TRAIL‐induced apoptosis unclear. Here, we report that ISG12a is localized to the mitochondria and nuclear membrane in HCC cells and promotes the translocation of NR4A1 from nucleus to cytoplasm. ISG12a augments TRAIL‐induced apoptosis by activating the intrinsic apoptotic pathway. We propose that activation of NF‐κB mediates NR4A1 induction by TRAIL in TRAIL‐resistant hepatoma cells. Inhibition of NF‐κB activation reversed TRAIL‐induced NR4A1 induction. NR4A1 knockdown was able to overcome TRAIL resistance in hepatocellular carcinoma cells.

## 2 MATERIALS AND METHODS

### 2.1 Cell culture

Tumour necrosis factor‐related apoptosis‐inducing ligand‐resistant liver cancer cell line Huh7 and TRAIL sensitive liver cancer cell line LH86 were used in this study as previously described.^17^, ^18^ Human embryonic kidney cells HEK293T were cultured in DMEM with 10% foetal bovine serum at 37°C and 5% CO_2_.

### 2.2 Antibodies and reagents

Antibodies against Noxa, Puma, Bax, Bax 6A7 and protein A/G beads were obtained from Santa Cruz Biotechnology (Santa Cruz, CA, USA). Lamin B1, ISG12a antibodies were from Abcam. Caspase‐9, caspase‐3, poly (ADP‐ribose) polymerase (PARP), NR4A1, H3, tubulin and β‐actin antibodies were from Cell Signalling Technology (Beverly, MA, USA). IκBα, p‐JNK, Jun N‐terminal kinases (JNK), GAPDH and Tubulin antibodies were from Abclonal. Bcl2‐BH3 was from ABGENT. green fluorescent protein (GFP) antibody was from Abbkine. Flag‐M2 antibody was from Sigma. Lipofectamine 2000, Mitotracker and 4',6‐diamidino‐2‐phenylindole were obtained from Invitrogen. BAY11‐7082 and SP600125 were purchased from MedChem Express. Tumour necrosis factor‐related apoptosis‐inducing ligand reagent was obtained from R&D Company (Minneapolis, MN, USA). Annexin V/Propidium Iodide (PI) apoptosis detection kit was from Keygentic (Nanjing, China).

### 2.3 Western blot analysis

Cell lysates were prepared as previously described,^18^ and equal amounts of protein were resolved by SDS‐PAGE, transferred onto polyvinylidene fluoride membrane (Millipore, Bedford, MA, USA) and probed with appropriate dilutions of primary and secondary antibodies. The immunoreactive protein complexes were visualized by chemiluminescence by using the West Pico system (Thermo, Rockford, IL, USA).

### 2.4 Immunoprecipitation assay

Cells were lysed in NP40 buffer (50 mmol/L Tris‐HCl, pH7.5, 150 mmol/L NaCl, 0.5% NP‐40 and 50 mmol/L NaF) containing cocktail protease inhibitors. Lysate was incubated with primary antibody at 4°C for 12 hours, and then protein A/G‐sepharose beads were added at 4°C for 4 hours incubation. Beads were harvested by centrifugation. After washing thrice with NP40 buffer, beads were boiled in 40 μL loading buffer and analysed by Western blot.

### 2.5 Immunofluorescence assay

For immunofluorescence assay, cells were plated on glass slides for overnight and sequentially treated in accordance with corresponding experimental conditions. Then cells were fixed with 3.7% Para‐formaldehyde and permeabilized with permeabilizing solution (0.2% [W/V] Triton X‐100 in Phosphate buffered saline [PBS]). After blocking with 5% bovine serum albumin C at room temperature for 30 minutes, cells were incubated with specific antibodies and the fluorescence was detected by confocal microscopy (Zesis).

### 2.6 Plasmids and siRNA transfection

Flag‐tagged full length ISG12a constructs used in this study have been previously described.^17^ The nine‐nucleotide deletion variant cDNA of ISG12a (ISG12a‐S) was purchased from Vigene, and then, Flag‐tagged ISG12a‐S was cloned into a p3XFLAG‐CMV‐14 vector. Enhanced green fluorescent protein (EGFP)‐tagged ISG12a‐F was cloned into pEGFP‐N1 vector. NR4A1 cDNA was also purchased from Vigene, Flag‐tagged and EGFP‐tagged NR4A1 were then cloned into p3XFLAG‐CMV‐14 and pEGFP‐N3 vector respectively. MtDsRed plasmid was kindly provided by Dr Yong Liu (Cancer Institute, XuZhou Medical University). All constructs were confirmed by direct DNA sequencing.

The siRNA sequence for NR4A1 (5′‐GGCUUGAGCUGCAGAAUG AdTdT‐3′) was previously reported.^19^ The siRNA sequences for ISG12a were 5′‐CUGCAGAAGAGAACCAUTT‐3′ and 5′‐GAGGAUCUCUUACUCUCUATT‐3′. The control siRNA sequence is 5′‐UUCUCCGAACGUGUCACGUTT‐3′. These siRNA oligonucleotides targeting NR4A1 and ISG12a and control oligonucleotides were synthesized by Genepharma (Shanghai, China). All the overexpressing plasmids and interfering small interference RNA (siRNA) were transfected into cells using Lipofectamine 2000 according to the manufacturer's protocol.

To obtain a high knockdown efficiency, 50 nmol/L siRNA was used. Cells were transfected with siRNA or plasmids for 48 hours and then treated with TRAIL or inhibitors at indicated times. Unless otherwise stated, 20 ng/mL TRAIL was used to treat HCC cells.

### 2.7 Annexin V/PI assay

Cells were seeded in six‐well plates and cultured overnight. After treatment with 10 ng/mL TRAIL for 3 hours, cells were collected and washed with PBS, and then incubated with Annexin V/PI for 15 minutes before Fluorescence‐activated cell sorting (FACS) analysis.

### 2.8 Subcellular fractionation

Subcellular fractionations were prepared as described previously.^19^ Briefly, cells were suspended in 0.5 mL cold buffer A (10 mmol/L 4‐(2‐Hydroxyethyl) piperazine‐1‐ethanesulfonic acid‐potassium hydroxide (HEPES‐KOH), pH 7.9, 1.5 mmol/L MgCl_2_, 10 mmol/L KCl and 0.5 mmol/L dithiothreitol (DTT)) and homogenized. Cell extracts were centrifuged at 14 000 *g* for 30 seconds, and the cytoplasmic fraction was then collected. The nuclei pellets were resuspended in cold high‐salt buffer C (20 mmol/L HEPES‐KOH, pH 7.9, 25% glycerol, 420 mmol/L NaCl, 1.5 mmol/L MgCl_2_, 0.2 mmol/L ethylenediaminetetraacetic acid and 0.5 mmol/L DTT) containing proteinase cocktail inhibitors on ice for 30 minutes. The lysates were centrifuged at 14 000 *g* at 4°C for 15 minutes, and the nuclear fraction was then collected.

## 3 RESULTS

### 3.1 ISG12a is localized to the mitochondria and nucleus in liver cancer cells

**Figure 1:**
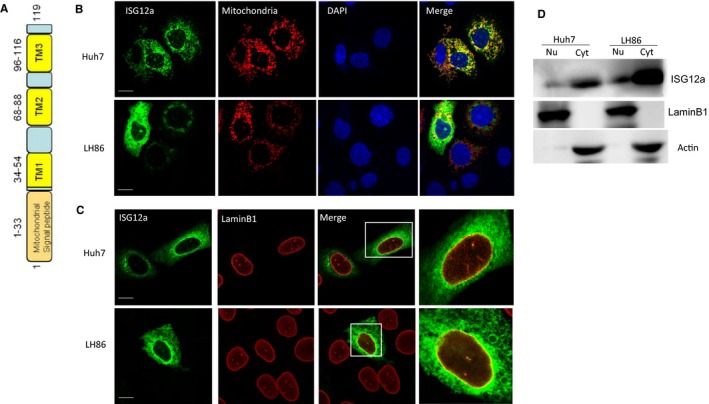
Localization of ISG12a in hepatoma cells. A, Schematic representation of ISG12a protein with putative mitochondria signal peptide and predicted transmembrane domains shown. B, Immunofluorescence staining of Flag‐tagged ISG12a in Huh7 and LH86 cells. Huh7 and LH86 cells were stained with anti‐flag (Green) and 4',6‐diamidino‐2‐phenylindole (DAPI) (Blue). mtDsRed plasmid was transfected into Huh7 and LH86 cells as a mitochondrial marker. Scale bar, 20 µm. C, ISG12a localized to the nucleus. Cells were transfected with Flag‐tagged ISG12a plasmid and stained with anti‐flag (Green), anti‐Lamin B1 (Red) and DAPI (Blue). Immunostained samples were imaged using confocal microscope. Scale bars, 20 µm. D, Western blot analysis of nuclear (Nu) and cytoplasmic (Cyt) fractionation of endogenous ISG12a in Huh7 andLH86 cells. Lamin B1 and actin were used as nuclear and cytoplasmic fraction markers, respectively

To identify the subcellular localization of full length ISG12a in liver cancer cells, we first analysed the protein sequence of ISG12a using UniProtKB software. ISG12a protein modelling identified an N‐terminal mitochondrial matrix‐targeting signal peptide and three transmembrane domains, thereby suggesting that it localizes to the mitochondria and is a membrane protein (Figure 1A). To assess its precise subcellular localization in liver cancer cells, Huh7 and LH86 cells were transiently co‐transfect with Flag‐tagged ISG12a and mtDsRed plasmids. Its localization was assessed by confocal microscopy 48 hours post‐transfection. As shown in Figure 1B, ISG12a was distributed in the mitochondria. To further investigate whether ISG12a localizes to the nucleus, Huh7 and LH86 cells were transiently transfected with Flag‐tagged ISG12a. At 48 hours post‐transfection, cells were fixed and stained with inner nuclear envelope marker Lamin B1. ISG12a co‐localized with Lamin B1 (Figure 1C), thereby indicating that ISG12a is also localized to nucleus. Consistent with the immunofluorescence results, cell fractionation studies also showed that the majority of ISG12a was in the cytoplasm and a minor fraction accumulates within the nucleus (Figure 1D). These results indicate that ISG12a is localized to the mitochondria and nucleus in liver cancer cells.

### 3.2 ISG12a enhances TRAIL‐induced apoptosis though the intrinsic apoptotic pathway

**Figure 2:**
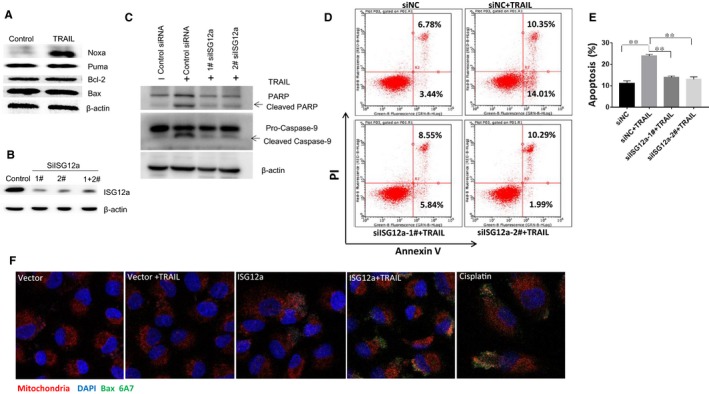
ISG12a increases tumour necrosis factor‐related apoptosis‐inducing ligand (TRAIL) activity through mitochondrial‐dependent apoptotic pathway. A, Western blot analysis of mitochondrial‐dependent apoptotic pathway‐related proteins in LH86 cells upon TRAIL stimulation. B, Western blot analysis to confirm ISG12a knockdown in LH86 cells transfected with different small interference RNA (siRNA) for 48 h. C, LH86 cells were transfected with ISG12a siRNAs for 48 h followed by TRAIL treatment, poly (ADP‐ribose) polymerase and caspase‐9 were examined by Western blot. β‐actin was used as loading control. D, LH86 cells were transfected with control or ISG12a siRNA for 48 h followed by 10 ng/mL TRAIL treatment for 3 h. Annexin V/Propidium Iodide (PI) assay was performed to detect cell apoptosis. E, Quantitation of apoptotic cells. F, Huh7 cells were transfected with vector or ISG12a for 48 h and treated with TRAIL for 3 h. Cisplatin (10 µg/mL) was used as positive control. Then cells were stained with Mitotracker (red), bcl‐2‐associated X protein 6A7 (green) and 4',6‐diamidino‐2‐phenylindole (blue). Scale bars, 20 µm

We next determined whether ISG12a increased TRAIL activity through intrinsic apoptotic pathway according to its mitochondrial localization. We treated LH86 cells with 10 ng/mL TRAIL for 3 hours and then examined several mitochondrial protein expressions by Western blot, because our previous studies have shown that ISG12a is highly expressed in LH86 cells.^17^ As shown in Figure 2A, Noxa, a regulator of mitochondrial‐dependent apoptosis, was robustly induced in LH86 cells after TRAIL treatment. To further confirm that ISG12a promotes TRAIL‐induced apoptosis through intrinsic apoptotic pathway in TRAIL‐sensitive HCC cells, LH86 cells were transfected with ISG12a‐targeting siRNAs for 48 hours and treated with TRAIL for 3. Caspase‐9 and PARP cleavage were attenuated after ISG12a knock‐down in LH86 cells (Figure 2B). Flow cytometry assay also showed that knock down of ISG12a reduced TRAIL‐induced apoptosis in LH86 cells, thereby further confirming that ISG12a augmented TRAIL activity (Figure 2D,E). Bax is a major proapoptotic member of Bcl2 family required at the decisional stage of apoptosis. Previous study has demonstrated that TRAIL‐induced mitochondrial‐dependent apoptosis requires Bax activation.^20^ Activated Bax exerts 6A7 conformational change and translocates to mitochondria after cellular stress.^21^ To test whether Bax was activated in TRAIL‐resistant cells in the presence of ISG12a, Huh7 cells were transfected with ISG12a‐FLAG (a protein tag) plasmids and treated with TRAIL. We then examined activated Bax by means of suitable conformation‐specific Bax 6A7 antibody by immunofluorescence assay. TRAIL treatment activated higher Bax activity by promoting its 6A7 conformational change in ISG12a‐ overexpressing Huh7 cells compared with vector expression cells (Figure 2F). These results suggested that ISG12a augments TRAIL‐induced apoptosis in hepatoma cells through mitochondrial‐dependent apoptotic pathway.

### 3.3 ISG12a promotes nuclear export and cytoplasm migration of NR4A1

**Figure 3:**
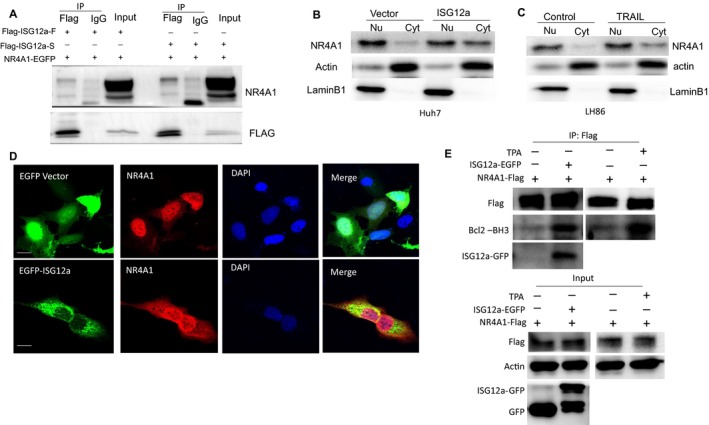
A, Immunoprecipitation analysis of the interaction between ISG12a and NR4A1. 293T cells were co‐transfected with NR4A1‐Enhanced green fluorescent protein (EGFP) and full length ISG12a‐Flag (ISG12a‐F) or short isoform ISG12a (ISG12a‐S). Cell lysates were immunoprecipitated by using monoclonal anti‐Flag M2 antibody. Lysates and immunoprecipitates were examined by immunoblotting using anti‐Flag and anti‐NR4A1 antibodies. B, ISG12a reduced the presence of NR4A1 in nucleus. Huh7 cells were transfected with ISG12a‐Flag as indicated. The amount of NR4A1 was assessed in cytosolic (Cyt) and nuclear (Nu) fractions by immunoblot analysis, and laminB1 and actin were used as nuclear and cytosolic protein controls, respectively. C, Tumour necrosis factor‐related apoptosis‐inducing ligand (TRAIL) induces NR4A1 translocation to the cytoplasm. LH86 cells were treated with 10 ng/mL TRAIL and the amount of NR4A1 was assessed in nuclear and cytosolic fractions by immunoblot analysis. D, Immunocytochemistry was used to analyse cellular localization of NR4A1 in the absence or presence of ISG12a. Huh7 cells were co‐transfected with ISG12a‐EGFP or vector with Flag‐NR4A1. Then cells were stained with anti‐Flag (red) and 4',6‐diamidino‐2‐phenylindole (blue). Scale bars, 20 µm. E, Huh7 cells were co‐transfected with Flag‐NR4A1 and EGFP‐ISG12a or EGFP vector for 48 h and then treated with TRAIL. TPA (100 ng/mL) was used as the positive control. Cell lysates were immunoprecipitated by using monoclonal anti‐Flag M2 antibody. Lysates and immunoprecipitates were examined by immunoblotting using Flag, Bcl2 BH3 and green fluorescent protein antibodies

To test whether NR4A1 is involved in ISG12a‐mediated TRAIL activity, we performed immunoprecipitation experiments to confirm the interaction between ISG12a and NR4A1. EGFP‐tagged NR4A1 interacted both with Flag‐tagged full‐length ISG12a and a short isoform of ISG12a, with nine‐nucleotide deletions in exon 4 and have stronger apoptosis induction ability, thereby suggesting that the transmembrane 1 domain of ISG12a might not be necessary for the interaction with NR4A1 (Figure 3A). To identify the cellular function of the interaction between ISG12a and NR4A1, we transfected Flag‐tagged ISG12a in Huh7 cells and then measured NR4A1 distribution. As shown in Figure 3B, in the presence of overexpressed ISG12a, the amount of cytosolic NR4A1 increased. In LH86 cells highly expressing ISG12a, TRAIL treatment resulted in an increase in cytosolic NR4A1 expression (Figure 3C). To further confirm that ISG12a mediates nuclear export of NR4A1, the subcellular distribution of overexpressed FLAG‐NR4A1 was examined by immunofluorescence assay, in the presence of ISG12a. EGFP‐tagged NR4A1 is mainly located in nucleus in vector transfected cells (Figure 3D). However, overexpression of ISG12a in Huh7 cells reduced nuclear localization of NR4A1. Interestingly, cytosolic NR4A1 increased and co‐localized with ISG12a in ISG12a‐overexpressing Huh7 cells (Figure 3D, bottom panel). The interaction and co‐localization in cytoplasm of NR4A1 with ISG12a indicated that NR4A1 might target the mitochondria. A previous study has reported that mitochondrial NR4A1 converts Bcl2 from a cytoprotective to a cytodestructive molecule by promoting Bcl2 to exposure its BH3 domain.^22^ To test whether ISG12a‐mediated NR4A1 cytoplasm migration affect Bcl2 conformation change, we immunoprecipitated NR4A1 in Huh7 cells transfected with EGFP‐ISG12a and Flag‐NR4A1 and then examined the converted Bcl2 using Bcl2‐BH3 antibody that can specially recognize a conformational change in Bcl2's BH3 domain.^22^ As shown in Figure 3E, ISG12a overexpression markedly increased Bcl2 conformation change that exposure its BH3 domain compared with control cells. Bcl2 is a main factor that confers resistance to TRAIL. Thus, these results therefore suggested that ISG12a‐mediated NR4A1 cytoplasm migration and downstream Bcl2 conformation change enhance TRAIL activity in liver cancer cells.

### 3.4 TRAIL induces nuclear accumulation of NR4A1 in TRAIL‐resistant cells

**Figure 4:**
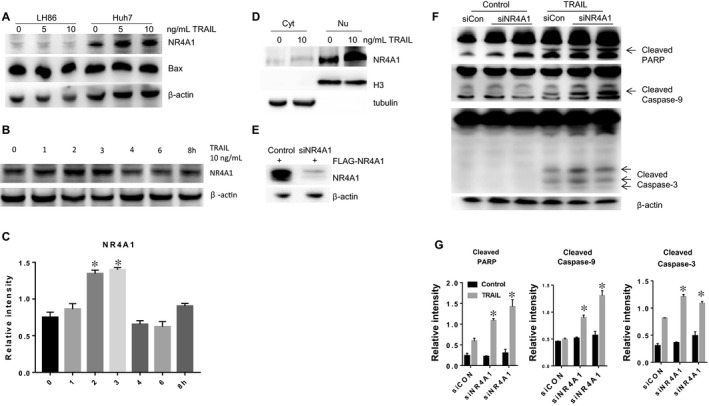
A, LH86 and Huh7 cells were treated with tumour necrosis factor‐related apoptosis‐inducing ligand (TRAIL) for 3 h at indicated concentrations. NR4A1, bcl‐2‐associated X protein and β‐actin proteins were detected by Western blot assay. B, Huh7 cells were treated with 10 ng/mL TRAIL for indicated times. NR4A1 expression was examined by Western blotting assay. C, Quantitation of NR4A1. Data represent the mean ± SEM of three independent experiments from immunoblot analysis. *P < 0.05; **P < 0.01; ***P < 0.001. D, Huh7 cells were treated with TRAIL for 3 h. The amount of NR4A1 was assessed in cytosolic and nuclear fractions by immunoblot analysis. E, Huh7 cells were transfected with control or NR4A1 siRNA for 48 h. Western blot analysis was used to confirm the siRNA interference efficiency. F, Knockdown of NR4A1 sensitized Huh7 cells to TRAIL. Huh7 cells were transfected with control or NR4A1 siRNA for 48 h, followed by 10 ng/mL TRAIL treatment, poly (ADP‐ribose) polymerase (PARP), caspase‐9 and‐3 were examined by Western blot. G, Quantitation of cleaved PARP, Caspase 9 and Caspase 3. Data represent the mean ± SEM of three independent experiments from immunoblot analysis. *P < 0.05; **P < 0.01; ***P < 0.001

Next, we determined whether NR4A1 alone had some effects on TRAIL‐induced apoptosis in HCC cells, because a recent study reported that TNF‐α rapidly induces NR4A1 expression in breast cancer cells.^19^ To test whether TRAIL has a similar ability to induce NR4A1 expression in hepatoma cells, we treated LH86 and Huh7 cells with TRAIL and examined NR4A1 expression by Western blot. As shown in Figure 4A, TRAIL induced NR4A1 expression in a dose‐dependent manner only in TRAIL‐resistant Huh7 cells. Time course analysis results indicated that the induction of NR4A1 by TRAIL occurred after 2 hours of treatment and gradually decreased 3 hours after TRAIL treatment (Figure 4B). We also observed that the basal expression level of NR4A1 in LH86 cells is lower than in Huh7 cells (Figure 4A). The survival effects of NR4A1 depended on its nuclear localization.^23^ These results suggest that highly expressed NR4A1 might confer TRAIL resistance to hepatoma cells. To determine whether NR4A1 is associated with TRAIL resistance, TRAIL‐treated Huh7 cells were fractionated into nuclear and cytoplasmic extracts, and NR4A1 localization was detected by immunoblot analysis. Results in Figure 4C showed that TRAIL‐induced NR4A1 protein mainly resided in the nucleus. To determine the biological function of NR4A1, Huh7 cells were transfected with NR4A1 siRNA for 48 hours and treated with TRAIL for 3 hours. As shown in Figure 4E, knockdown of NR4A1 increased TRAIL‐induced PARP cleavage and caspas‐9 and ‐3 activation. Collectively, these results indicated that highly expressed NR4A1 conferred TRAIL resistance and TRAIL‐mediated NR4A1 induction might further increase this resistance.

### 3.5 TRAIL induces NR4A1 expression partly through NF‐κB pathway

**Figure 5:**
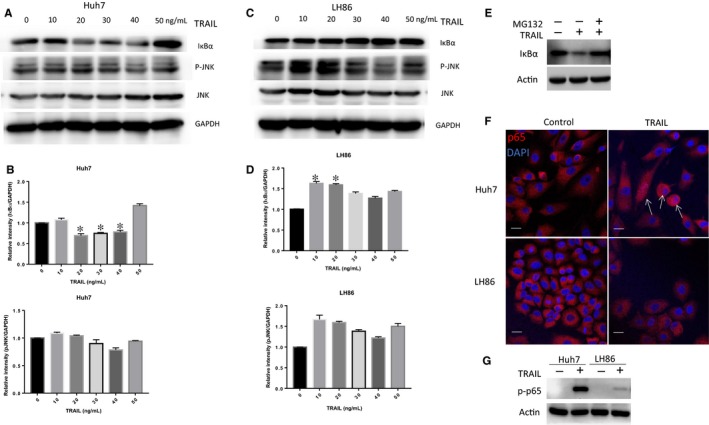
(A,C) Huh7 and LH86 cells were treated with tumour necrosis factor‐related apoptosis‐inducing ligand (TRAIL) for 3 h at indicated concentrations, cell extracts were detected by Western blot with antibodies against IκBα, pJNK, Jun N‐terminal kinases (JNK) and GAPDH. (B,D) Quantitation of immunoblot. Data represent the mean ± SEM of three independent experiments from immunoblot analysis. *P < 0.05; **P < 0.01; ***P < 0.001. E, Huh7 cells were pre‐treated with 20 µmol/L MG132 or vehicle for 1 h, followed by 20 ng/mL treatment for 3 h. The IκBα level was examined by Western blot analysis. F, Huh7 and LH86 cells were treated with 20 ng/mL for 3 h and then stained with p65 and 4',6‐diamidino‐2‐phenylindole (DAPI). The white arrows highlight the nuclear localization of p65 in the immunofluorescence stain as well as the DAPI counterstain. G, Huh7 and LH86 cells were treated with 20 ng/mL for 3 h and then the phosphorylation of p65 was examined by Western blot analysis. H, Huh7 cells were pre‐treated with vehicle or NF‐κB inhibitor Bay‐11‐7082 (10µmol/L) for 1 h, followed by treatment with 20 ng/mL TRAIL for 3 h. Cell extracts were examined by Western blotting. I, LH86 cells were pre‐treated with vector control or JNK inhibitor for 1 h followed by 20 ng/mL TRAIL for 3 h. Cell extracts were examined by Western blot

Lastly, we investigated the pathways that might be involved in NR4A1 induction under TRAIL treatment in liver cancer cells. It was previously reported that TRAIL‐induced activation of NF‐κB pathway was responsible for HCC cells resistance to TRAIL‐induced apoptosis.^24^ Another study demonstrated that TRAIL can induce apoptosis through activation of JNK in HCC cells.^25^ According to the abovementioned studies, we have been suggested that TRAIL may activate different pathways in TRAIL‐sensitive and TRAIL‐resistant cells. To test this hypothesis, we treated Huh7 and LH86 cells with various concentrations of TRAIL and detected NF‐κB and JNK pathway activity by Western blot analysis. As shown in Figure 5A and B, 20 ng/mL TRAIL treatment was sufficient to induce IκBα degradation, but did not affect JNK in Huh7 cells. In contrast, TRAIL could activate JNK but not NF‐κB in LH86 cells (Figure 5C,D). Moreover, TRAIL‐mediated IκBα degradation in Huh7 cells was almost completely diminished by the treatment of MG132 (Figure 5E). To further confirm that TRAIL selectively activated NF‐κB in resistant liver cells, we treated Huh7 and LH86 cells with 20 ng/mL TRAIL for 3 hours and examined p65 translocation by immunofluorescence assay. TRAIL treatment resulted in nuclear translocation of p65 in Huh7 cells but not in LH86 cells (Figure 5F). In addition, phosphorylated p65 was markedly increased in Huh7 cells upon TRAIL treatment (Figure 5G). To test whether activated NF‐κB and JNK pathways were related to TRAIL‐induced NR4A1 induction, Huh7 and LH86 cells were pre‐treated with NF‐κB or JNK inhibitor for 1 hour respectively, and then treated with TRAIL for 3 hours. NF‐κB inhibition by BAY11‐7082 partly reversed TRAIL‐induced NR4A1 expression in Huh7 cells (Figure 5F). Intriguingly, we observed that TRAIL slightly induced NR4A1 expression when JNK was inhibited in LH86 cells (Figure 5G). These results indicated that HCC cells were resistant to TRAIL partly due to NF‐κB pathway‐mediated NR4A1 induction. However, in TRAIL‐sensitive HCC cells, TRAIL treatment activated JNK instead of NF‐κB.

## 4 DISCUSSION

To date numerous clinical trials using TRAIL‐based therapies have been conducted. There is an ongoing phase 2 clinical trial that study the DR4 agonist Mapatumumab in combination with Sorafenib in subject with advanced hepatocellular carcinoma.^26^ However, due to the resistance of many primary cancer cells to monotherapy with TRAIL‐R agonists, as well as the lack of biomarkers to identify patients who responded to these agents, the anticancer activities of TRAIL‐based agents have exhibited limited clinical benefit. Although our previous study has demonstrated that ISG12a is highly expressed in TRAIL‐sensitive hepatoma cells and enhances TRAIL‐induced apoptosis, the downstream mechanisms by which ISG12a increases TRAIL activity still remain unclear. In this study, we found that ISG12a was localized to the mitochondria and nuclear envelope in liver cancer cells, although a most recent study has reported that ISG12a and several of its variants are only localized to the mitochondria.^11^ Thus, the localization of ISG12a might be variable and dependent on cell context. The mitochondrial localization of ISG12a suggests that it increases the cytotoxic effects of TRAIL through intrinsic apoptotic pathway. We found decreased caspase‐9 activity in LH86 cells with ISG12a knockdown. In contrast, when ISG12a was overexpressed in TRAIL‐resistant Huh7 cells, TRAIL treatment induced Bax conformation change, thereby activating mitochondrial‐dependent apoptosis. These findings together with results of our previous studies,^17^ suggested that ISG12a might serve as a biomarker and a new therapeutic target for HCC patients with TRAIL‐based therapies.

**Figure 6:**
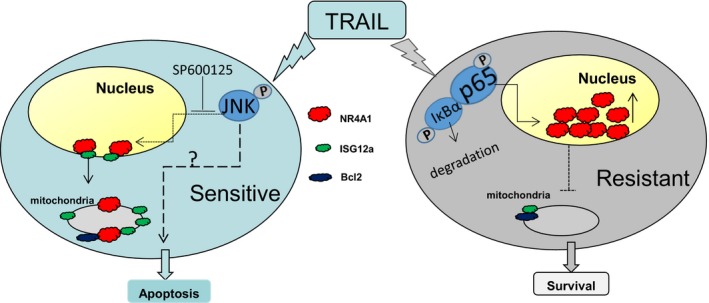
Schematic representation for how ISG12a and NR4A1 regulate tumour necrosis factor‐related apoptosis‐inducing ligand (TRAIL) activity in TRAIL‐sensitive and TRAIL‐resistant HCC cells. In TRAIL‐sensitive cells, highly expressed ISG12a mainly localizes to the mitochondria and mediates TRAIL‐induced apoptosis through intrinsic apoptotic pathway. Nuclear‐localized IGS12a promotes NR4A1 translocation to the cytoplasm. Cytosolic NR4A1 interacts with ISG12a and may form a complex with Bcl2 to regulate TRAIL‐induced apoptosis. Jun N‐terminal kinases (JNK) activation may be associated with TRAIL‐induced apoptosis. Inhibition of JNK leads to the increase in NR4A1 expression upon TRAIL treatment. In TRAIL‐resistant cells, TRAIL induces NR4A1 expression through activation of NF‐kB. Increased NR4A1 accumulates in the nucleus and may exert prosurvival functions under TRAIL treatment

The orphan nuclear receptor NR4A1 was identified as a novel interaction partner of ISG12a. ISG12a inactivates the vasculoprotective functions of NR4A1 by inhibiting its transcriptional activity.^15^ We confirmed the interaction between ISG12a and NR4A1 in HCC cells. Our results also showed that ISG12a overexpression resulted in NR4A1 migration from nucleus to cytoplasm and downstream Bcl2 conformation change. Previous studies have reported that Bcl2 could interact both with ISG12a and NR4A1,^11^, ^22^, ^27^ thus, these three molecules might form complexes and affect functions of the mitochondria (Figure 6). Notably, the short isoform of ISG12a also interacted with NR4A1. ISG12a‐S has a stronger ability to induce apoptosis than the full length ISG12a.^11^ Thus, the interaction between ISG12a‐S and NR4A1 might be more potent in regulating mitochondrial‐dependent apoptosis upon TRAIL stimulation and only exist in TRAIL‐sensitive cells. ISG12a‐S may offer a novel TRAIL response biomarker with important clinical implications.

NR4A1 has a paradoxical role in diverse cancer types. NR4A1 was identified as an oncogenic driver in pancreatic, renal, lung and colon cancers.^28^, ^29^ Several other studies have reported that NR4A1 was a tumour suppressor in lymphoma, triple‐negative breast cancer and hepatocellular carcinoma.^32^, ^33^ The expression and nuclear accumulation of NR4A1 confer chemotherapeutic resistance in some types of cancer. For example, fenretinide induces apoptosis of Huh7 cells through NR4A1 induction and cytoplasmic translocation, whereas fenretinide could not induce apoptosis of HepG2 cells seemingly due to NR4A1 accumulation in the nucleus.^35^ Tumour necrosis factor‐α‐induced NR4A1 expression and nuclear accumulation reduce its apoptotic effects in breast cancer cells.^19^ In our study, we found that NR4A1 was highly expressed in TRAIL‐resistant HCC cells. Tumour necrosis factor‐related apoptosis‐inducing ligand treatment further induces NR4A1 expression and nuclear accumulation. Knockdown of NR4A1 could overcome TRAIL resistance in Huh7 cells.

Apart from inducing apoptosis, binding of TRAIL to its receptors can also activate NF‐κB pathway to promote cell proliferation.^36^, ^37^ We also observed TRAIL‐induced activation of NF‐κB in Huh7 cells. Inhibition of NF‐κB activity by specific inhibitors decreased TRAIL‐mediated NR4A1 induction. We also found that TRAIL can activate JNK in LH86 cells. Inhibition of JNK activity increases TRAIL‐induced NR4A1 expression. These results indicate that TRAIL might exert its death effect through JNK activation in TRAIL‐sensitive HCC cells. However in TRAIL‐resistant liver cancer cells, TRAIL‐induced NR4A1 expression through NF‐κB activation may attenuate its death induction effect.

In summary, our study demonstrates that ISG12a is localized to the mitochondria and nucleus in hepatoma cells. ISG12a augments TRAIL‐induced intrinsic apoptotic pathway activation through its mitochondria localization and interaction with NR4A1. NR4A1 was highly expressed in TRAIL‐resistant cells compared with TRAIL‐sensitive cells. TRAIL treatment activates NF‐κB and induces NR4A1 expression in resistant cells. In TRAIL‐sensitive cells, JNK pathway is activated after TRAIL stimulation. These findings suggest that ISG12a and NR4A1 may offer new TRAIL response markers and combining NF‐κB inhibiting agents with TRAIL‐based agonists may overcome TRAIL resistance.

## CONFLICT OF INTEREST

The authors declare that they have no competing interests.
